# Dos(e)Age: Role of Dose and Age in the Long-Term Effect of Cannabinoids on Cognition

**DOI:** 10.3390/molecules27041411

**Published:** 2022-02-19

**Authors:** Erica Zamberletti, Tiziana Rubino

**Affiliations:** Neuroscience Center, Department of Biotechnology and Life Sciences (DBSV), University of Insubria, 21052 Busto Arsizio, VA, Italy; tiziana.rubino@uninsubria.it

**Keywords:** cannabinoids, cognition, prenatal period, adolescence, aging, animal studies

## Abstract

Cannabis is still the most widely used illicit drug around the world. While its use has always been prevalent among adolescents, recent evidence suggests that its consumption is also increasing among other population groups, such as pregnant women and aged people. Given the known impact of cannabis on brain development and behavior, it is important to dissect the possible long-term impact of its use across different age groups, especially on measures of cognitive performance. Animal models of cannabinoid exposure have represented a fundamental tool to characterize the long-lasting consequences of cannabinoids on cognitive performance and helped to identify possible factors that could modulate cannabinoids effects in the long term, such as the age of exposure and doses administered. This scoping review was systematically conducted using PubMed and includes papers published from 2015 to December 2021 that examined the effects of cannabinoids, either natural or synthetic, on cognitive performance in animal models where exposure occurred in the prenatal period, during adolescence, or in older animals. Overall, available data clearly point to a crucial role of age in determining the long-term effect of cannabinoid on cognition, highlighting possible detrimental consequences during brain development (prenatal and adolescent exposure) and beneficial outcomes in old age. In contrast, despite the recent advances in the field, it appears difficult to clearly establish a possible role of dosage in the effects of cannabinoids on cognition, especially when the adolescent period is taken into account.

## 1. Introduction

### Trends in Cannabis Use across Ages

Cannabis is still one of the most widely used drug worldwide. Almost 4% of the global population aged between 15 and 64 years used cannabis at least once in the last year, according to the 2021 World Drug Report [1].

Δ9-Tetrahydrocannabinol (THC) is the main intoxicating psychoactive constituent present in the plant, while cannabidiol (CBD) is the most studied cannabinoid, which is likely to be devoid of THC-like psychoactive effects. In the past two decades, there have been rapid advances in cannabis cultivation techniques, particularly in Europe and North America, which are mainly aimed at achieving a high THC content. Hence, cannabis potency has increased by as much as four times in several parts of the world [1]. According to the report, the percentage of THC in cannabis seized increased from around 4% to 16% between 1995 and 2019 in the United States, while it almost doubled in Europe, from around 6% to more than 11% between 2002 and 2019 [1]. Daily cannabis use has been associated with a greater risk of developing psychotic disorders, and the likelihood of such disorders is greater among those people who use cannabis with a THC content ≥10% on a daily basis [2].

Prevalence and patterns of cannabis use vary greatly across age groups. In Europe, cannabis use in the past year among people aged 15–34 was particularly high, at an estimated 15% [3]. When only 15- to 24-year-olds are considered, the prevalence of cannabis use is higher, with 19% having used the drug in the last year. Annual teenage cannabis use in the United States has remained stable over the last few years, with around 13% of adolescents aged 12–17 years using cannabis, according to the Substance Abuse and Mental Health Services Administration [4]. While the number of teens using cannabis has not changed substantially, the frequency of use is increasing, with more teens using cannabis daily [1].

Outside of teens, older adults aged ≥65 years currently represent the fastest-growing population of cannabis users. Cannabis use by individuals aged 65 and older has increased more than eightfold in recent years, from less than 0.5% in 2006 to 4.2% in 2018 [5]. Multiple factors, such as reduced restrictions on possession and sale by many states, as well as the scientific evidence of supposed benefits of cannabis for many conditions which are predominant among this population, could likely be driving this trend. Although more population-based research is needed, it seems that the majority of older cannabis users take cannabis for medical purposes either as self-treatment or as prescribed medicine [6]. In line with this, older adults tend to select cannabis high in CBD content compared with young people who tend to select cannabis strains with the highest THC content [6,7].

Recently, an increased prevalence of cannabis use has also been described among pregnant women. Indeed, when comparing cannabis use among pregnant women in the United States between 2002 and 2017, data collected revealed that cannabis use has increased from 3.4% to 7.0% overall, regular daily cannabis use increased from 0.9% to 3.4% among pregnant women, and cannabis use was more common during the first trimester than during the second and third [8]. Unfortunately, the real prevalence of cannabis use among pregnant people in Europe is still difficult to establish, as available data are limited to single-country analysis [9,10]. Increased prevalence of cannabis use during pregnancy could be attributed to the changes in attitudes toward cannabis use, which might result in reductions in the perception of risk of regular cannabis use over time [11]. In addition, the potential use of cannabis for treating nausea and vomiting and other conditions common during pregnancy may explain the observed trends. Accordingly, a recent study showed that pregnant women with nausea and vomiting during pregnancy had nearly 2–4 times greater odds of prenatal cannabis use [12].

The increased prevalence of cannabis use among each of these populations should be approached with caution. Indeed, heavy cannabis use in teens has been frequently associated with functional and structural brain impairments negatively impacting attention, processing speed, motor coordination, verbal memory, and executive function [13]. Although these effects may be milder with use of lighter cannabis described in older adults, this population shows a greater susceptibility to cognitive decline, underscoring the need to examine factors (i.e., cannabis use) that may exacerbate age-related cognitive decline. Lastly, attention should be paid to possible detrimental effects of regular cannabis use during pregnancy on cognitive development and function of the child, which might be subtle at first and not be detectable for many months or even years after birth.

The increased prevalence of cannabis use reported in these sensitive population subgroups highlights the need to examine the effects of cannabis use on cognitive function across ages.

This review summarizes and critically discusses available studies in animal models which have dealt with the investigation of chronic cannabinoid exposure during gestation, during adolescence, or in older animals on measures of cognitive functions. The final aim of this review was to determine the current extent of the literature and identify gaps in knowledge regarding the effects of cannabinoid use on cognitive function across different ages.

## 2. Methodology

A scoping review of the literature was systematically conducted using PubMed. Papers published from 2015 to December 2021 were included if they examined the effects of cannabinoids, either naturally occurring or synthetic, on cognitive performance in animal models where exposure occurred in the prenatal period, during adolescence, or in older animals. Studies published before 2015 on these topics have been comprehensively and critically discussed elsewhere [14,15,16,17]. Search terms included a combination of the following: cannabinoid, THC, adolescen*, pubertal, prenatal, aging, elderly, and cognition. No additional filters were added to allow for maximum inclusiveness. Studies had to be in English, and only those examining long-term effects of chronic cannabinoids in animal models on behavioral outcomes related to cognitive performance were included. Studies that focused on human populations, employed acute treatments, did not assess cognitive performance, or conflated cannabinoids with other substances or other underlying susceptibility factors were excluded. Publications in this review did not include letters to the editor, review articles, or commentaries. Titles, abstracts, and eligible full-text publications were independently screened and separately evaluated by the authors (E.Z. and T.R.), blind to each other’s decisions. Disagreements were resolved through consensus following each review stage.

## 3. Results

**Figure 1:**
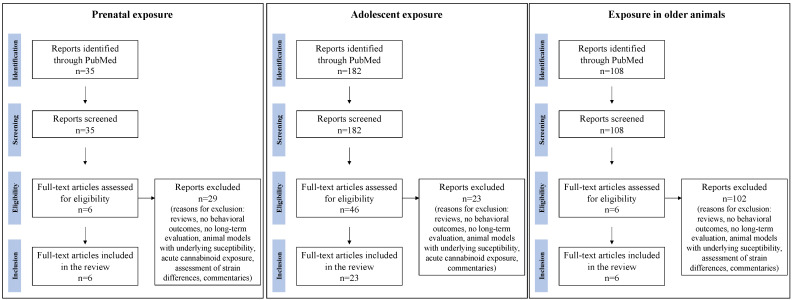
Academic database search of published literature between 2015 and December 2021 related to the long-term effects of cannabinoid exposure on cognition in the prenatal period, during adolescence, and in older animals.

The initial search resulted in 35 results for studies with prenatal exposure, 182 results for studies in adolescent animal models, and 108 results for studies in the elderly. A review of each published title and abstract was completed for select studies that were consistent with the inclusion and exclusion criteria, resulting in the selection of six articles for studies in animal models of prenatal exposure, 23 articles for studies in adolescent animal models, and six articles for studies in older models (Figure 1).

## 4. Discussion

### 4.1. Effects of Prenatal Cannabinoid Exposure on Cognition

Knowledge gained in the last 30 years has widely demonstrated that the endocannabinoid system is critically involved in brain plasticity and neural cell development, as well as neuronal differentiation and connectivity [18]. Since the prenatal brain developmental window is characterized by all these processes aimed at the formation of neuronal cells and networks, perturbation of the fine regulatory role of the endocannabinoid system through exposure to exogenous cannabinoids in this very period appears to be detrimental for brain health [16,19].

Several research groups have used animal models to thoroughly investigate the effect of prenatal exposure to cannabinoids on the cognitive performance of the offspring during adolescence or adulthood. Data coming from these studies reveal the presence of subtle but still measurable cognitive deficits after in utero cannabinoid exposure. The impairments have been described in spatial learning and memory, attention tasks, emotional memory, and short-term memory [17]. More recent papers are also in line with this assessment (Table 1), reporting alterations in spatial memory [20], short-term memory [21], aversive limbic memory [22], and behavioral flexibility [23] after prenatal THC exposure. The disruptive effect of prenatal THC was observed independently from the route of administration, being present when the drug was given orally [21], parenterally [20,22], or through vapor [23]. Interestingly, Weimar reported that only the cannabis vapor more enriched in THC (400 mg/mL vs. 50 mg/mL) was able to induce the flexibility impairment, suggesting the existence of a dose-dependent effect.

Curiously enough, a recent paper reported that fetal exposure to the synthetic cannabinoid WIN55,212-2 did not induce cognitive deficits in prepubertal and pubertal animals of both sexes [24], whereas previous studies applying the same protocol of exposure described impaired cognitive performance in the offspring [25,26].

Cognitive impairments observed after cannabinoid exposure during gestation were produced by treatments usually spanning from gestation day (GD) 5 to GD 20. However, memory impairments were also reported when THC was given during a more restrict period, i.e., from GD 10.5 to GD 17.5 [20], suggesting that this specific developmental window may represent the predominant prenatal period where the endocannabinoid system exerts its most delicate neurodevelopmental role. Indeed, CB1 receptor (CB1R) mRNA and receptor density gradually increase during fetal development starting from GD11 [27], exactly when neural tube formation occurs [28].

It is worth noting that, in rodents, the period after birth, more precisely the first week, actually represents the last period of gestation in humans [29]. Cannabinoid exposure of the rodent offspring through lactation during this period has been described to produce alterations in the motivational and social behavior of the progeny paralleled by alterations in prefrontal cortex synaptic function [30]; however, no data are available about their cognitive performance.

The mechanism through which cannabis may exert its detrimental effects on progeny cognition likely entails the disruption of the physiological role played by the endocannabinoid system during the prenatal sensitive developmental window. Accordingly, since the endocannabinoid tone is involved in the modulation of axonal growth and guidance, it is not surprising that THC may disrupt fetal brain connectivity, especially in the hippocampus and the cortex, the very neuronal networks underpinning memory encoding, cognition, and executive skills [31]. Moreover, prenatal THC also alters the glutamatergic, GABAergic, dopaminergic, opioidergic, and serotonergic systems [17,32], as well as cerebral levels of kynurenic acid, a neuroactive compound that can affect the extracellular levels of major neurotransmitters critically involved in cognitive processes [21]. A relevant role in complex cognitive functions is played by hippocampal and cortical GABAergic interneurons that are essential to coordinate firing of pyramidal neurons. Of note, endocannabinoids during prenatal brain development seem to be crucial for the proper interneuron placement and integration into neuronal networks; therefore, THC interference with these finely orchestrated processes impacts the excitatory/inhibitory balance [19]. Recent support for the prenatal THC-induced interneuronopathy theory was provided by de Salas-Quiroga and colleagues [20]. In addition, they demonstrated that the presence of aberrant circuitries caused by the persistent reduction in cholecystokinin-positive (CCK^+^) interneurons was restricted to the male progeny, suggesting the existence of a sex-dependent effect. Indeed, the long-term interneuronopathy, paralleled by altered hippocampal function and impaired spatial cognition, was described only in prenatal exposed males. In the same line, the prenatal THC-induced hyperdopaminergic state was also found to be male-selective [33]. These recent results point toward the necessity to evaluate both sexes when studying the impact of prenatal THC exposure.

Lastly, in addition to THC, another important component of cannabis is the non-psychoactive compound CBD, and many women report using CBD oil during pregnancy to reduce severe pregnancy-related nausea [34]. Research about the safety profile of CBD during pregnancy is still in its infancy. Indeed, only one paper has been published so far about the potential impact of CBD exposure during perinatal development [35]. The authors reported the association of developmental CBD exposure in mice with changes in the brain methylome and sex-specific effects on anxiety behavior and memory. Specifically, working spatial memory seems to be improved by prenatal CBD in the female progeny but not affected in males. These observations may be relevant when translated into humans, in which cannabis exposure always refers to exposure to a mixture of compounds, the most concentrated being THC and CBD. Accordingly, we can speculate that greater CBD content leads to less impact on the progeny, at least on cognition, and vice versa. In the future, further studies are needed with mixed compounds in animal models to better mimic the effect of cannabis exposure in humans.

**Table 1:** Summary of studies assessing lasting effects of prenatal cannabinoid exposure on cognition.

Article	Species and Sex	Dose and Delivery in Dams	Age of Behavioral Assessment	Outcome
[20]	mice; males and females	THC 3 mg/kg; intraperitoneal injections; E10.5–E17.5	P60–90	No alterations in the novel object recognition task; impaired performance in the object location task in male mice only
[21]	Wistar rats; males	THC 5 mg/kg; oral gavage; E5–E20	P65–90	Impaired short-term memory in the Y-maze test
[22]	Wistar rats; males	THC 2 mg/kg; subcutaneous injections; E5–E20	P25–30	No alterations in the novel object recognition task; impairments in the emotional object recognition test
[23]	Long Evans rats; males and females	400 mg/mL cannabis extract (99.2 mg/mL THC, 4.8 mg/mL CBD, and 8.4 mg/mL CBN) or 50 mg/mL cannabis extract; twice a day one-hour vapor exposure; mating and gestation	P80–P110	Impaired behavioral flexibility in the attentional set-shifting after 400 mg/mL cannabis extract in both sexes
[24]	Wistar rats; males and females	WIN55,212-2 0.5 mg/kg; subcutaneous injections; E5–E20	P28–P35 (males) P22–P28 (females); P50–60 (males) P30–P40 (females)	No alterations in the temporal order memory test at both periods in both sexes
[35]	mice; males and females	CBD 20 mg/kg daily; oral gavage; from 14 days before mating through gestation and lactation	P84	Improved performance in the Y-maze test in females; no alterations in males

### 4.2. Effects of Adolescent Cannabinoid Exposure on Cognition

Adolescence represents a crucial timing of neurodevelopment characterized by key structural and functional changes that are required for proper behavioral and cognitive maturation [36]. The adolescent brain is highly plastic and still immature with respect to the adult one, suggesting a major vulnerability to harmful environmental influences, such as drug use. As mentioned above, cannabis use is particularly prevalent among adolescents. The recent legalization in many countries reflects a generalized change in attitudes and beliefs toward cannabis. In this scenario, providing a reliable and clear scientific evidence on possible detrimental consequences of adolescent cannabis use on brain development and behavior is, therefore, of overwhelming importance. Of particular concern is the potential influence of cannabis on measures of cognition, given the ongoing maturation of neural circuits mediating complex cognitive processes during adolescence [37].

Lasting alterations in cognitive functions have been reported in rodents chronically exposed to cannabinoids during adolescence (Table 2). Impairments in working memory processes have been observed using object recognition and T-maze tasks in both male and female rodent models following adolescent but not adult treatment [38,39,40,41,42,43,44]. Chronic escalating doses of THC resulted in protracted defects on short-term novel object recognition memory when exposure occurred during early but not late adolescence [45]. In contrast, no long-term changes were found in hippocampal-dependent object location task, social recognition memory, and acquisition and reversal in the water T-maze [45]. These data suggest that the specific period of administration might be crucial in determining possible long-term cognitive changes triggered by THC and that adolescent THC exposure might have fewer long-term effects on cognitive task related to hippocampal activity. In line with this, the lack of detrimental effects of chronic THC exposure during adolescence on hippocampal-dependent pure spatial memory tasks has also been observed in other studies [46]. Hence, it could be possible that chronic exposure to cannabinoids during adolescence might have more marked effects on cognitive abilities involving frontal region functioning. Accordingly, enduring although subtle deficits on cognitive processes related to flexibility and decision making have been described using attentional set-shifting tasks and probabilistic reward choice tasks after administration of WIN55,212-2 in male and female rats [47], suggesting that chronic exposure to synthetic cannabinoids could adversely affect executive functions into adulthood. In contrast, lasting effects on attentional set-shifting performance were not reported after exposure to escalating low doses of THC during adolescence in both male and female rats, whereas the same low doses resulted in impaired spatial memory [48,49]. Long-term deficits in associative learning memory have also been reported, with rats exposed to THC during adolescence taking longer to learn a paired-associate learning task when tested in adulthood [50]. The adverse impact of adolescent THC exposure on working memory performance has also been demonstrated in nonhuman primates. Indeed, repeated THC exposure in adolescent male rhesus monkeys impaired the reinforcement-related learning processes required for improving performance on spatial working memory tasks [51]. Of note, these deficits were mitigated by an extended period of continued training [52], suggesting the possibility of potential interventions for preventing/relieving THC-induced working memory deficits and promoting protection/recovery of cognitive function. In this context, modulation of THC-induced cognitive defects by CBD has been recently investigated. Concomitant administration of THC and CBD in a ratio of 1 to adolescent male mice was shown to prevent the development of cognitive deficits at adulthood [40]. However, protection was not observed when a higher THC/CBD ratio was administered to adolescent female rats [53]. Similarly, administration of the same THC/CBD ratio failed to prevent deficits on cognitive measure of learning and cognitive flexibility in adolescent nonhuman primates [54]. The discrepant results obtained using different animal models and sexes do not allow to draw any conclusion on the possible protection mediated by CBD toward long-term cognitive deficits triggered by adolescent THC. Contrasting results have also been obtained when investigating the effects of the administration of CBD alone during adolescence on measures of cognitive performance. Chronic treatment with CBD in adolescent female rats led to the development of short-term memory deficits into adulthood [53], whereas no detrimental effects on cognition were reported when CBD was administered during adolescence to both male and female mice, even at higher doses [55]. These data highlight a different sensitivity to the effects of CBD on cognition and clearly demonstrate the need for a more careful investigation of long-term effects of CBD alone and in combination with THC in the future.

Long-term effects of adolescent cannabinoid exposure on cognition have recently been investigated in operant models of THC and cannabinoid self-administration. Adolescent WIN55,212-2 self-administration resulted in either improvements or no change in adult working memory performance in both male and female rats [56,57]. Similarly, working memory performance was unaltered or slightly improved in males only, after adolescent self-administration of THC [58]. Remarkably, studies employing self-administration paradigms did not highlight any long-term cognitive deficits following adolescent cannabinoid exposure, suggesting that volitional control of THC exposure, possibly associated with exposure to nonintoxicating doses, could have a less detrimental impact on cognitive performance in the long-term.

While all the above-cited studies employed systemic injections of cannabinoid agonists, the majority of cannabis use in humans occurs via inhalation, particularly smoking. However, to date, very few studies have investigated the long-term effects of cannabis or THC vapor inhalation on measures of cognition in preclinical models. Exposure to cannabis smoke or ascending doses of THC vapor during adolescence did not affect novel object recognition memory in rats when assessed at adulthood [59]. Similarly, chronic exposure to cannabis smoke in adolescent rats did not impact cognitive flexibility in adulthood in either set-shifting or probabilistic reversal learning tasks [60]. Overall, unlike what has been widely demonstrated using experimenter administration protocols, cannabinoid exposure under conditions of intravenous and smoke self-administration in adolescent models seems to have less detrimental effects on cognitive performance in the long term. However, the absence of cognitive impairments resulting from intravenously self-administered or smoked cannabinoids should not be manipulated to convey the wrong message that adolescent cannabis use is harmless, as subjective effects of cannabinoids can greatly differ among species. Animal models could likely display a different control over drug intake with respect to humans, making it difficult to establish whether doses that are voluntarily self-administered by animals could reflect those that are abused by humans.

### 4.3. Effects of Cannabinoid Exposure in Older Animals

Aging is a physiological process that cannot be stopped but needs to be controlled to achieve healthy aging. Cognitive decline is an integral aspect of aging, and it appears as a spontaneous and continuous process due to changes occurring in specific brain areas. In some cases, these changes are magnified by the emergence of neurodegenerative conditions such as Alzheimer’s disease (AD).

Physiological aging appears to be paralleled by changes in the brain endocannabinoid system [61]. Specifically, in older animals, a decline in the expression and activity of CB1R has been reported, together with alterations in the levels of synthetic and degrading enzymes. Interestingly, in CB1 knockout mice, an accelerated cognitive decline, paralleled by neuronal loss and chronic inflammation, has been described, thus supporting the notion that CB1R signaling may play a role in the control of the aging process.

According to this hypothesis, the paucity of papers investigating the effect of modulation of the endocannabinoid system in aged rodents seems to suggest that replenishing the lost endocannabinoid signaling would be beneficial to slowing down the physiological aging process (Table 3) [62,63,64]. Low doses of THC have been employed to reach this goal, i.e., to improve the cognitive performance of aged mice. Interestingly, while Bilkei-Gorzo [62] and Nidadavolu [63] obtained this result after chronic treatment, Sarne [64] demonstrated the positive effect of a single ultralow dose of THC (0.002 mg/kg) in aged female mice that lasted at least 7 weeks. They suggested that a single dose of THC was able to induce long-lasting structural alterations, likely through the involvement of Sirtuin-1, a protein deacetylase that was shown to be involved in synaptic plasticity, memory formation, learning capability, neuronal development, and neuroprotection, and that was found decreased in old mice [64].

These results are strikingly in contrast with those observed in young rodents, where chronic THC administration usually impairs cognitive performance, thus suggesting the existence of an age-dependent effect. In addition, Nidadavolu and colleagues [63] reported another interesting age-dependent response; while in young mice the co-administration of a combination of THC and CBD seems to be able to prevent some of the detrimental THC effect on cognition, in aged mice, this combination did not restore the memory impairment caused by physiological aging, suggesting that THC alone is more efficacious in the recovery effect. The inability of a CBD/THC mixture to reduce the cognitive impairment present in old mice (the wildtype controls) was also observed by Aso et al. [65] when investigating the CBD/THC effect in a transgenic mouse model of AD. In this context, however, chronic administration of a combination of THC and CBD resulted effective in improving cognitive performance disrupted by the presence of β-amyloid, even when administered at advanced stages of the disease [65]. The cognitive improvement was paralleled by changes in markers of synaptic function relevant for the recovery of the imbalance in excitatory vs. inhibitory neural activity observed in the cortex of aged transgenic mice. Lastly, when administered at an early symptomatic stage, chronic exposure to the combination of THC and CBD reduced in a more efficient way the learning impairment and induced a more marked reduction in gliosis than THC or CBD separately in mice with AD-like pathology [66]. As a whole, these data suggest that CBD might not be effective in restoring the memory impairment induced by physiological aging, but could represent an add-on compound whenever in the presence of intense neurodegeneration, such as during AD progression.

Although the above-cited papers point toward a paramount role of CB1R signaling in slowing down the age-associated cognitive decline, some recent papers also suggest a possible involvement of CB2 receptors (CB2R), especially when considering “inflammaging”, which is the idea that aging is associated with a progressive decline in the ability to cope with stressors and a progressive increase in the whole-body load of proinflammatory cytokines [67]. In other studies [68,69], the authors explored the beneficial effect of a natural CB2R agonist, β-caryophyllene, toward age-related cognitive decline in mice. Interestingly, this compound was able to reverse the deficit in working memory and higher levels of the inflammatory cytokine IL-23 observed in aged mice [68], as well as the detrimental changes in astrocytes and DNA oxidation present in the d-galactose animal model of induced aging [69].

In conclusion, despite research on cannabinoid effects in elderly still being in its infancy, results obtained so far clearly suggest that low-dose treatment with THC could be a potential strategy to slow down or even reverse age-dependent cognitive decline. The mechanisms of this positive outcome could be associated with activation of both CB1R and CB2R, likely under-stimulated in the elderly brain due to age-dependent changes in the endocannabinoid system.

**Table 3:** Summary of studies assessing effects of cannabinoid exposure on cognition in aged animals.

Article	Species and Sex	Dose and Delivery	Age	Behavioral Outcome
[62]	C57BL6/J mice; males	THC (3 mg/kg/day); Alzet minipumps implanted subcutaneously with a delivery of 28 days	12 and 18 months	Improved performance in the Morris water maze, the novel object location recognition task and the partner recognition task
[63]	C57BL6/J mice; males	THC (1 mg/kg/day), or a 1:1 mixture of THC and CBD (THC/CBD, 1 mg/kg/day each); Alzet minipumps implanted subcutaneously with a delivery of 28 days	18 months	1 mg/kg/day THC dose improved spatial learning in the Morris water maze;1:1 combination of THC and CBD had no effect
[64]	Institute of Cancer Research mice; females	THC 0.002 mg/kg; single intraperitoneal injection	24 months	Better performance in 6 different behavioral assays of various aspects of memory and learning
[65]	AβPP/PS1 transgenic mice; males	THC 0.75 mg/kg + CBD 0.75 mg/kg; intraperitoneal injections once a day for 5 weeks	12 months	Reduced memory impairment in the two-object recognition test in a V-maze
[68]	Swiss-Webster mice; males	β-caryophyllene 100 and 178 mg/kg; intraperitoneal injections 3 days a week for one week	12 months	Improved performance in the Y-maze task
[69]	BALB/c mice; males	β-caryophyllene 10 mg/kg; oral administration for 4 weeks	12 weeks (ageing induced by 8 weeks of treatment with D-galactose)	No effect on cognitive flexibility in the Morris water maze test

## 5. Conclusions

Animal models of cannabinoid exposure represent a unique tool to characterize the long-lasting behavioral consequences of cannabinoids on cognitive performance, and to identify possible factors that could modulate cannabinoids effects in the long term. Indeed, very different results can be obtained on the basis of the different cannabinoid used, doses, animal models, strains, and sexes, as well as the cognitive tasks employed. However, the most striking factor in predicting the outcome of cannabinoid use seems to be age. Indeed, what is detrimental during brain development (prenatal and adolescent exposure) has been reported to be beneficial in old age. This might be related to the decline in endocannabinoid tone observed in aging. It is well known that the endocannabinoid system works as a buffer system to maintain homeostasis in the brain; thus, its decline may have a negative impact on brain health, accelerating the aging process. In contrast, despite the recent advances in the field, it appears difficult to clearly establish a possible role of dosage in the effects of cannabinoids on cognition, particularly when administration occurs during the adolescent period. Indeed, cannabinoids administered during adolescence can have long-term effects on specific cognitive tasks; however, the appearance and persistence of such effects vary greatly depending on the paradigm of administration and behavioral task used, making it impossible to draw any definitive conclusion.

### Future Directions

Despite the increasing use of cannabis among pregnant women and older people, there are currently few studies that have specifically investigated the long-term effect of cannabinoids when administered in these specific stages, if compared to the extensive investigations carried out in animal models of adolescent exposure. Future investigations are, therefore, needed to explore more thoroughly the possible consequences of cannabis consumption within these populations, especially using operant models of cannabinoid exposure, such as self-administration paradigms and vapor inhalation. In addition, given the paucity of studies, future research should also be aimed at better dissecting possible long-term effects of CBD, either alone or in combination with THC, on cognitive performance, especially when administered at early stages of brain development. Concerning adolescent exposure to cannabinoids and its impact on cognition, additional studies should be carried out to provide a scientific explanation for the discrepant findings that have been reported when comparing the effects of passive experimenter administration protocols with intravenously self-administered or inhaled cannabinoids. Unlike what has been reported during developmental phases, the positive effects of cannabinoids on cognition observed in older animals suggest the need for further studies employing more suitable formulations and routes of administration in light of a possible therapeutic exploitation of cannabinoids in this specific population.

## Figures and Tables

**Table 2:** Summary of studies assessing lasting effects of adolescent cannabinoid exposure on cognition.

Article	Species and Sex	Dose and Delivery	Age	Outcome
[38]	Sprague-Dawley rats; females	THC 2.5–5–10 mg/kg twice a day; intraperitoneal injections	P35–P45	Impaired performance in the T-maze
[39]	Inbred C57Bl/6J and DBA/2J mice; males	THC 10 mg/kg intraperitoneal injections every 72 h	P28–P44	Impaired performance in the object recognition test
		THC 10 mg/kg intraperitoneal injections every 72 h	P69–P85	No impairment in the object recognition test
[40]	CD1 mice; males	THC 3 mg/kg, CBD 3 mg/kg, CBD + THC 3 mg/kg each; daily intraperitoneal injections	P28–P48	Impairment in the object recognition memory after THC; counteracted by concomitant CBD
[41]	Sprague-Dawley rats; females	THC 2.5–5–10 mg/kg twice a day; intraperitoneal injections	P35–P45	Impaired performance in the object recognition test
[42]	Sprague-Dawley rats; females	THC 2.5–5–10 mg/kg twice a day; intraperitoneal injections	P35–P45	Impaired performance in the object recognition test
[43]	Sprague-Dawley rats; males	THC 2.5–5–10 mg/kg twice a day; intraperitoneal injections	P35–P45	Impaired performance in the object recognition test
[44]	Sprague-Dawley rats; females	THC 2.5–5–10 mg/kg twice a day; intraperitoneal injections	P35–P45	Impaired performance in the object recognition test
[45]	Sprague-Dawley rats; males	THC 2.5–5–10 mg/kg twice a day; intraperitoneal injections	P30–P41	Impaired performance in the object recognition test
[46]	Long-Evans rats; males	THC 3–5–10 mg/kg; subcutaneous injections	P30–P45	No impairment in the Barnes maze test
[47]	Long-Evans rats; males and females	WIN55,212-2 1.2 mg/kg; intraperitoneal injections	P30–P60	Impairment in the probabilistic reward choice task in both sexes
[48]	Sprague-Dawley rats; males and females	THC 0.3–1–3 mg/kg twice a day; intraperitoneal injections	P35–P45	Impaired spatial memory in the object location task
[49]	Sprague-Dawley rats; males	THC 0.3–1–3 mg/kg twice a day; intraperitoneal injections	P35–P45	Impaired spatial memory in the Morris water maze
[50]	Long-Evans rats; males	THC 2.5–5–10 mg/kg twice a day; intraperitoneal injections	P35–P45	Impairment in paired-associate learning task
[51]	Rhesus monkeys; males	THC 15 to 240 μg/kg 5 days/week for 12 months; intravenous injections	24–36 months	Impaired the reinforcement-related learning processes
[52]	Rhesus monkeys; males	THC 15 to 240 μg/kg 5 days/week for 12 months; intravenous injections	24–36 months	Impaired reinforcement-related learning processes were mitigated after protracted training
[53]	Sprague-Dawley rats; females	THC 2.5–5–10 mg/kg twice a day; intraperitoneal injections	P35–P45	Impaired performance in the object recognition test
		THC/CBD 3:1 ratio		Impaired performance in the object recognition test
		CBD 5 mg/kg twice a day		Impaired performance in the object recognition test
[54]	Squirrel monkeys; males	THC (0.1–1 mg/kg) for 3 weeks; daily injections	Adolescence; age range not specified	Impaired discrimination learning, no effect on cognitive flexibility.
		THC + CBD 1:3 (0.1–1:0.3–3 mg/kg) for 3 weeks;daily injections		CBD did not modulate THC effects on cognitive performance
[55]	C57BL/6J mice; males and females	CBD 20 mg/kg twice a day; intraperitoneal injections	P25–P45	No effects on spatial memory in the Barnes Maze; improved learning in the task
[56]	Sprague-Dawley rats; females	WIN55,212-2 0.0125 mg/kg/infusion; intravenous self-administration (fixed ratio 1)	P34–P59	No lasting deficits in the object location test and delayed-match-to-sample working memory task
[57]	Sprague-Dawley rats; males	WIN55,212-2 0.0125 mg/kg/infusion; self-administration (fixed ratio 1)	P38–P49	No lasting deficits in the object memory test and object location test
[58]	Sprague-Dawley rats; males and females	THC 3–10–30–100 μg/kg/infusion intravenous self-administration (fixed ratio 1)	P32–P51	Unaltered performance in the delayed-match-to-sample working memory task; enhanced working memoryperformance in males that self-administered high doses of THC
[59]	Long-Evans rats; males and females	5.6% THC, 0% cannabidiol and 0.4% cannabinol; cannabis smoke	P29–P49	No effects in the novel object recognition task
		THC 2.5–5–10 mg/kg twice a day; intraperitoneal injections	P35–P45	No effects in the novel object recognition task
[60]	Long-Evans rats; males	5.6% THC, 0% cannabidiol and 0.4% cannabinol; cannabis smoke	P29–P49	No effects on cognition (set shifting, reversal learning, intertemporal choice)

## Data Availability

Not applicable.

## Sample Availability

Samples of the compounds are not available from the authors.

## References

[1] United Nations World Drug Report 2021.

[2] Di Forti M., Quattrone D., Freeman T.P., Tripoli G., Gayer-Anderson C., Quigley H., Rodriguez V., Jongsma H.E., Ferraro L., La Cascia C. (2019). The contribution of cannabis use to variation in the incidence of psychotic disorder across Europe (EU-GEI): A multicentre case-control study. Lancet Psychiatry.

[3] European Monitoring Centre for Drugs and Drug Addiction (2020). European Drug Report 2020: Key Issues Summary.

[4] U.S. Department of Health and Human Services, Substance Abuse and Mental Health Services Administration, Center for Behavioral Health Statistics and Quality (2018). National Survey on Drug Use and Health 2016 (NSDUH-2016-DS0001). https://www.datafiles.samhsa.gov/dataset/national-survey-drug-use-and-health-2016-nsduh-2016-ds0001.

[5] Han B.H., Palamar J.J. (2020). Trends in Cannabis Use Among Older Adults in the United States, 2015–2018. JAMA Intern. Med..

[6] Choi N.G., DiNitto D.M., Marti C.N. (2018). Older marijuana users’ marijuana risk perceptions: Associations with marijuana use patterns and marijuana and other substance use disorders. Int. Psychogeriatr..

[7] Sexton M., Cuttler C., Mischley L.K. (2019). A Survey of Cannabis Acute Effects and Withdrawal Symptoms: Differential Responses Across User Types and Age. J. Altern. Complement. Med..

[8] Volkow N.D., Han B., Compton W.M., McCance-Katz E.F. (2019). Self-reported Medical and Nonmedical Cannabis Use Among Pregnant Women in the United States. JAMA.

[9] Lozano J., Garcia-Algar O., Marchei E., Vall O., Monleon T., Di Giovannandrea R., Pichini S. (2007). Prevalence of gestational exposure to cannabis in a Mediterranean city by meconium analysis. Acta Paediatr..

[10] Roncero C., Valriberas-Herrero I., Mezzatesta-Gava M., Villeg4eas J.L., Aguilar L., Grau-Lopez L. (2020). Cannabis use during pregnancy and its relationship with fetal developmental outcomes and psychiatric disorders. A systematic review. Reprod. Health.

[11] Odom G.C., Cottler L.B., Striley C.W., Lopez-Quintero C. (2020). Perceived Risk of Weekly Cannabis Use, Past 30-Day Cannabis Use, and Frequency of Cannabis Use Among Pregnant Women in the United States. Int. J. Womens Health.

[12] Young-Wolff K.C., Sarovar V., Tucker L.Y., Avalos L.A., Conway A., Armstrong M.A., Goler N. (2018). Association of Nausea and Vomiting in Pregnancy with Prenatal Marijuana Use. JAMA Intern. Med..

[13] Cyrus E., Coudray M.S., Kiplagat S., Mariano Y., Noel I., Galea J.T., Hadley D., Devieux J.G., Wagner E. (2021). A review investigating the relationship between cannabis use and adolescent cognitive functioning. Curr. Opin. Psychol..

[14] Rubino T., Parolaro D. (2016). The Impact of Exposure to Cannabinoids in Adolescence: Insights from Animal Models. Biol. Psychiatry.

[15] Levine A., Clemenza K., Rynn M., Lieberman J. (2017). Evidence for the Risks and Consequences of Adolescent Cannabis Exposure. J. Am. Acad. Child Adolesc. Psychiatry.

[16] Alpár A., Di Marzo V., Harkany T. (2015). At the Tip of an Iceberg: Prenatal Marijuana and Its Possible Relation to Neuropsychiatric Outcome in the Offspring. Biol. Psychiatry.

[17] Higuera-Matas A., Ucha M., Ambrosio E. (2015). Long-term consequences of perinatal and adolescent cannabinoid exposure on neural and psychological processes. Neurosci Biobehav. Rev..

[18] Lu H.C., Mackie K. (2021). Review of the Endocannabinoid System. Biol. Psychiatry Cogn. Neurosci. Neuroimaging.

[19] Zamberletti E., Rubino T. (2020). Impact of Endocannabinoid System Manipulation on Neurodevelopmental Processes Relevant to Schizophrenia. Biol. Psychiatry Cogn. Neurosci. Neuroimaging.

[20] De Salas-Quiroga A., García-Rincón D., Gómez-Domínguez D., Valero M., Simón-Sánchez S., Paraíso-Luna J., Aguareles J., Pujadas M., Muguruza C., Callado L.F. (2020). Long-term hippocampal interneuronopathy drives sex-dimorphic spatial memory impairment induced by prenatal THC exposure. Neuropsychopharmacology.

[21] Beggiato S., Ieraci A., Tomasini M.C., Schwarcz R., Ferraro L. (2020). Prenatal THC exposure raises kynurenic acid levels in the prefrontal cortex of adult rats. Prog. Neuro-Psychopharmacol. Biol. Psychiatry.

[22] Brancato A., Castelli V., Lavanco G., Marino R.A.M., Cannizzaro C. (2020). In utero Δ9-tetrahydrocannabinol exposure confers vulnerability towards cognitive impairments and alcohol drinking in the adolescent offspring: Is there a role for neuropeptide Y?. J. Psychopharmacol..

[23] Weimar H.V., Wright H.R., Warrick C.R., Brown A.M., Lugo J.M., Freels T.G., McLaughlin R.J. (2020). Long-term effects of maternal cannabis vapor exposure on emotional reactivity, social behavior, and behavioral flexibility in offspring. Neuropharmacology.

[24] Manduca A., Servadio M., Melancia F., Schiavi S., Manzoni O.J., Trezza V. (2019). Sex-specific behavioural deficits induced at early life by prenatal exposure to the cannabinoid receptor agonist WIN55, 212-2 depend on mGlu5 receptor signalling. J. Cereb. Blood Flow Metab..

[25] Antonelli T., Tomasini M.C., Tattoli M., Cassano T., Tanganelli S., Finetti S., Mazzoni E., Trabace L., Steardo L., Cuomo V. (2005). Prenatal Exposure to the CB1 Receptor Agonist WIN 55,212-2 Causes Learning Disruption Associated with Impaired Cortical NMDA Receptor Function and Emotional Reactivity Changes in Rat Offspring. Cereb. Cortex.

[26] Mereu G., Fà M., Ferraro L., Cagiano R., Antonelli T., Tattoli M., Ghiglieri V., Tanganelli S., Gessa G.L., Cuomo V. (2003). Prenatal exposure to a cannabinoid agonist produces memory deficits linked to dysfunction in hippocampal long-term potentiation and glutamate release. Proc. Natl. Acad. Sci. USA.

[27] Berrendero F., Romero J., Garcıa-Gil L., Suarez I., De la Cruz P., Ramos J., Fernández-Ruiz J. (1998). Changes in cannabinoid receptor binding and mRNA levels in several brain regions of aged rats. Biochim. Biophys. Acta Mol. Basis Dis..

[28] Scheyer A.F., Melis M., Trezza V., Manzoni O.J. (2019). Consequences of Perinatal Cannabis Exposure. Trends Neurosci..

[29] Hagberg H., Ichord R., Palmer C., Yager J.Y., Vannucci S.J. (2002). Animal Models of Developmental Brain Injury: Relevance to Human Disease. Dev. Neurosci..

[30] Scheyer A.F., Borsoi M., Pelissier-Alicot A.-L., Manzoni O.J. (2020). Maternal Exposure to the Cannabinoid Agonist WIN 55,12,2 during Lactation Induces Lasting Behavioral and Synaptic Alterations in the Rat Adult Offspring of Both Sexes. Eneuro.

[31] Tortoriello G., Morris C.V., Alpar A., Fuzik J., Shirran S., Calvigioni D., Keimpema E., Botting C.H., Reinecke K., Herdegen T. (2014). Miswiring the brain: 9-tetrahydrocannabinol disrupts cortical development by inducing an SCG10/stathmin-2 degradation pathway. EMBO J..

[32] Bara A., Ferland J.-M.N., Rompala G., Szutorisz H., Hurd Y.L. (2021). Cannabis and synaptic reprogramming of the developing brain. Nat. Rev. Neurosci..

[33] Frau R., Miczán V., Traccis F., Aroni S., Pongor C., Saba P., Serra V., Sagheddu C., Fanni S., Congiu M. (2019). Prenatal THC exposure produces a hyperdopaminergic phenotype rescued by pregnenolone. Nat. Neurosci..

[34] Sarrafpour S., Urits I., Powell J., Nguyen D., Callan J., Orhurhu V., Simopoulos T., Viswanath O., Kaye A.D., Kaye R.J. (2020). Considerations and Implications of Cannabidiol Use During Pregnancy. Curr. Pain Headache Rep..

[35] Wanner N.M., Colwell M., Drown C., Faulk C. (2021). Developmental cannabidiol exposure increases anxiety and modifies genome-wide brain DNA methylation in adult female mice. Clin. Epigenet..

[36] Spear L.P. (2000). Neurobehavioral changes in adolescence. Curr. Dir. Psychol. Sci..

[37] Luna B., Garver K.E., Urban T.A., Lazar N.A., Sweeney J.A. (2004). Maturation of cognitive processes from late childhood to adulthood. Child Dev..

[38] Cuccurazzu B., Zamberletti E., Nazzaro C., Prini P., Trusel M., Grilli M., Parolaro D., Tonini R., Rubino T. (2018). Adult Cellular Neuroadaptations Induced by Adolescent THC Exposure in Female Rats Are Rescued by Enhancing Anandamide Signaling. Int. J. Neuropsychopharmacol..

[39] Kasten C.R., Zhang Y., Boehm S.L. (2017). Acute and long-term effects of Delta 9-tetrahydrocannabinol on object recognition and anxiety-like activity are age- and strain-dependent in mice. Pharmacol. Biochem. Behav..

[40] Murphy M., Mills S., Winstone J., Leishman E., Wager-Miller J., Bradshaw H., Mackie K. (2017). Chronic Adolescent Delta(9)-Tetrahydrocannabinol Treatment of Male Mice Leads to Long-Term Cognitive and Behavioral Dysfunction, Which Are Prevented by Concurrent Cannabidiol Treatment. Cannabis Cannabinoid.

[41] Prini P., Rusconi F., Zamberletti E., Gabaglio M., Penna F., Fasano M., Battaglioli E., Parolaro D., Rubino T. (2018). Adolescent THC exposure in female rats leads to cognitive deficits through a mechanism involving chromatin modifications in the prefrontal cortex. J. Psychiatr. Neurosci..

[42] Rubino T., Prini P., Piscitelli F., Zamberletti E., Trusel M., Melis M., Sagheddu C., Ligresti A., Tonini R., Di Marzo V. (2015). Adolescent exposure to THC in female rats disrupts developmental changes in the prefrontal cortex. Neurobiol. Dis..

[43] Zamberletti E., Gabaglio M., Grilli M., Prini P., Catanese A., Pittaluga A., Marchi M., Rubino T., Parolaro D. (2016). Long-term hippocampal glutamate synapse and astrocyte dysfunctions underlying the altered phenotype induced by adolescent THC treatment in male rats. Pharmacol. Res..

[44] Zamberletti E., Gabaglio M., Prini P., Rubino T., Parolaro D. (2015). Cortical neuroinflammation contributes to long-term cognitive dysfunctions following adolescent delta-9-tetrahydrocannabinol treatment in female rats. Eur. Neuropsychopharmacol..

[45] Sabran-Cohen T., Bright U., Zer-Aviv T.M., Akirav I. (2021). Rapamycin prevents the long-term impairing effects of adolescence Delta-9-tetrahydrocannabinol on memory and plasticity in male rats. Eur. J. Neurosci..

[46] Nelson N.G., Law W.X., Weingarten M.J., Carnevale L.N., Das A., Liang N.C. (2019). Combined Delta(9)-tetrahydrocannabinol and moderate alcohol administration: Effects on ingestive behaviors in adolescent male rats. Psychopharmacology.

[47] Jacobs-Brichford E., Manson K.F., Roitman J.D. (2019). Effects of chronic cannabinoid exposure during adolescence on reward preference and mPFC activation in adulthood. Physiol. Behav..

[48] Poulia N., Delis F., Brakatselos C., Lekkas P., Kokras N., Dalla C., Antoniou K. (2020). Escalating low-dose Delta(9)-tetrahydrocannabinol exposure during adolescence induces differential behavioral and neurochemical effects in male and female adult rats. Eur. J. Neurosci..

[49] Poulia N., Delis F., Brakatselos C., Polissidis A., Koutmani Y., Kokras N., Dalla C., Politis P.K., Antoniou K. (2021). Detrimental effects of adolescent escalating low-dose Delta(9)-tetrahydrocannabinol leads to a specific bio-behavioural profile in adult male rats. Brit. J. Pharmacol..

[50] Abela A.R., Rahbarnia A., Wood S., Le A.D., Fletcher P.J. (2019). Adolescent exposure to Delta 9-tetrahydrocannabinol delays acquisition of paired-associates learning in adulthood. Psychopharmacology.

[51] Mathai D., Verrico C.D., Sampson A.R., Lewis D.A. (2018). The Effects of Repeated Delta-9-Tetrahydrocannabinol Administration to Adolescent Rhesus Monkeys on Working Memory. Am. J. Addict..

[52] Verrico C.D., Mathai D.S., Gu H., Sampson A.R., Lewis D.A. (2020). Recovery from impaired working memory performance during chronic Delta-9-tetrahydrocannabinol administration to adolescent rhesus monkeys. J. Psychopharmacol..

[53] Gabaglio M., Zamberletti E., Manenti C., Parolaro D., Rubino T. (2021). Long-Term Consequences of Adolescent Exposure to THC-Rich/CBD-Poor and CBD-Rich/THC-Poor Combinations: A Comparison with Pure THC Treatment in Female Rats. Int. J. Mol. Sci..

[54] Withey S.L., Kangas B.D., Charles S., Gumbert A.B., Eisold J.E., George S.R., Bergman J., Madras B.K. (2021). Effects of daily delta9-Tetrahydrocannabinol (THC) alone or combined with cannabidiol (CBD) on cognition-based behavior and activity in adolescent nonhuman primates. Drug Alcohol Depend..

[55] Kaplan J.S., Wagner J.K., Reid K., McGuinness F., Arvila S., Brooks M., Stevenson H., Jones J., Risch B., McGillis T. (2021). Cannabidiol Exposure During the Mouse Adolescent Period Is Without Harmful Behavioral Effects on Locomotor Activity, Anxiety, and Spatial Memory. Front. Behav. Neurosci..

[56] Kirschmann E.K., McCalley D.M., Edwards C.M., Torregrossa M.M. (2017). Consequences of Adolescent Exposure to the Cannabinoid Receptor Agonist WIN55,212-2 on Working Memory in Female Rats. Front. Behav. Neurosci..

[57] Kirschmann E.K., Pollock M.W., Nagarajan V., Torregrossa M.M. (2017). Effects of Adolescent Cannabinoid Self-Administration in Rats on Addiction-Related Behaviors and Working Memory. Neuropsychopharmacology.

[58] Stringfield S.J., Torregrossa M.M. (2021). Intravenous self-administration of delta-9-THC in adolescent rats produces long-lasting alterations in behavior and receptor protein expression. Psychopharmacology.

[59] Bruijnzeel A.W., Knight P., Panunzio S., Xue S., Bruner M.M., Wall S.C., Pompilus M., Febo M., Setlow B. (2019). Effects in rats of adolescent exposure to cannabis smoke or THC on emotional behavior and cognitive function in adulthood. Psychopharmacology.

[60] Hernandez C.M., Orsini C.A., Blaes S.L., Bizon J.L., Febo M., Bruijnzeel A.W., Setlow B. (2021). Effects of repeated adolescent exposure to cannabis smoke on cognitive outcomes in adulthood. J. Psychopharmacol..

[61] Di Marzo V., Stella N., Zimmer A. (2014). Endocannabinoid signalling and the deteriorating brain. Nat. Rev. Neurosci..

[62] Bilkei-Gorzo A., Albayram O., Draffehn A., Michel K., Piyanova A., Oppenheimer H., Dvir-Ginzberg M., Rácz I., Ulas T., Imbeault S. (2017). A chronic low dose of Δ9-tetrahydrocannabinol (THC) restores cognitive function in old mice. Nat. Med..

[63] Nidadavolu P., Bilkei-Gorzo A., Krämer M., Schürmann B., Palmisano M., Beins E.C., Madea B., Zimmer A. (2021). Efficacy of Δ9 -Tetrahydrocannabinol (THC) Alone or in Combination With a 1:1 Ratio of Cannabidiol (CBD) in Reversing the Spatial Learning Deficits in Old Mice. Front. Aging Neurosci..

[64] Sarne Y., Toledano R., Rachmany L., Sasson E., Doron R. (2018). Reversal of age-related cognitive impairments in mice by an extremely low dose of tetrahydrocannabinol. Neurobiol. Aging.

[65] Aso E., Andrés-Benito P., Ferrer I. (2016). Delineating the Efficacy of a Cannabis-Based Medicine at Advanced Stages of Dementia in a Murine Model. J. Alzheimer’s Dis..

[66] Aso E., Sánchez-Pla A., Vegas-Lozano E., Maldonado R., Ferrer I. (2014). Cannabis-Based Medicine Reduces Multiple Pathological Processes in AβPP/PS1 Mice. J. Alzheimer’s Dis..

[67] Franceschi C., Bonafè M., Valensin S., Olivieri F., De Luca M., Ottaviani E., De Benedictis G. (2000). Inflamm-aging: An evolutionary perspective on immunosenescence. Ann. N. Y. Acad. Sci..

[68] Lindsey L.P., Daphney C.M., Oppong-Damoah A., Uchakin P.N., Abney S.E., Uchakina O.N., Khusial R.D., Akil A., Murnane K.S. (2019). The cannabinoid receptor 2 agonist, β-caryophyllene, improves working memory and reduces circulating levels of specific proinflammatory cytokines in aged male mice. Behav. Brain Res..

[69] Chávez-Hurtado P., González-Castañeda R.E., Beas-Zarate C., Flores-Soto M.E., Viveros-Paredes J.M. (2020). β-Caryophyllene Reduces DNA Oxidation and the Overexpression of Glial Fibrillary Acidic Protein in the Prefrontal Cortex and Hippocampus of d-Galactose-Induced Aged BALB/c Mice. J. Med. Food.

